# RESPECT-ED: **R**ates of Pulmonary **E**mboli (PE) and **S**ub-Segmental **PE** with Modern **C**omputed **T**omographic Pulmonary Angiograms in **E**mergency **D**epartments: A Multi-Center Observational Study Finds Significant Yield Variation, Uncorrelated with Use or Small PE Rates

**DOI:** 10.1371/journal.pone.0166483

**Published:** 2016-12-05

**Authors:** David Mountain, Gerben Keijzers, Kevin Chu, Anthony Joseph, Catherine Read, Gabriel Blecher, Jeremy Furyk, Chrianna Bharat, Karthik Velusamy, Andrew Munro, Kylie Baker, Frances Kinnear, Ahses Mukherjee, Gina Watkins, Paul Buntine, Georgia Livesay, Daniel Fatovich

**Affiliations:** 1 Discipline of Emergency Medicine, School of Primary, Aboriginal and Rural Health Care, University of Western Australia, Perth, Australia; 2 Emergency Department, Sir Charles Gairdner Hospital, Perth, Western Australia, Australia; 3 Emergency Medicine Department, Gold Coast University Hospital, Southport, Queensland, Australia; 4 Department of Emergency Medicine, Royal Brisbane and Women's Hospital, Brisbane, Australia; 5 School of Medicine, University of Queensland, Brisbane, Queensland, Australia; 6 Emergency Medicine, Royal North Shore Hospital, Sydney, New South Wales, Australia; 7 Pleural Medicine Unit, Institute for Respiratory Health, Perth, Western Australia, Australia Centre for Respiratory Health, School of Medicine & Pharmacology, University of Western Australia, Perth, Western Australia, Australia; 8 Respiratory Medicine Unit (Research, Pleural Diseases) Sir Charles Gairdner Hospital, Perth, Western Australia, Australia; 9 Emergency Medicine, Monash Health, Melbourne, Victoria, Australia; 10 Department of Medicine, Monash University, Melbourne, Victoria, Australia; 11 Emergency Department, The Townsville Hospital, Townsville, Queensland, Australia; 12 Statistical Support, Department of Research, Sir Charles Gairdner Hospital, Perth, Western Australia, Australia; 13 Centre for Applied Statistics, University of Western Australia, Perth, Western Australia, Australia; 14 Emergency Department, Nelson Hospital, Nelson, New Zealand; 15 Emergency Medicine, Ipswich Hospital, Ipswich, Queensland, Australia; 16 Emergency Medicine, The Prince Charles Hospital, Brisbane, Queensland, Australia; 17 Emergency Medicine, Armadale General Hospital, Perth, Western Australia, Australia; 18 Emergency Medicine, Sutherland Hospital and Community Health Centres, Caringbah, Australia; 19 Emergency Department, Box Hill Hospital, Melbourne, Victoria, Australia; 20 Emergency Medicine Research, Princess Alexandra Hospital, Brisbane, Queensland, Australia; 21 Centre for Clinical Research in Emergency Medicine, Harry Perkins Institute of Medical Research, University of Western Australia, Perth, Australia; 22 Emergency Department, Royal Perth Hospital, Perth, Australia; Universite de Bretagne Occidentale, FRANCE

## Abstract

**Introduction:**

Overuse of CT Pulmonary Angiograms (CTPA) for diagnosing pulmonary embolism (PE), particularly in Emergency Departments (ED), is considered problematic. Marked variations in positive CTPA rates are reported, with American 4–10% yields driving most concerns. Higher resolution CTPA may increase sub-segmental PE (SSPE) diagnoses, which may be up to 40% false positive. Excessive use and false positives could increase harm vs. benefit. These issues have not been systematically examined outside America.

**Aims:**

To describe current yield variation and CTPA utilisation in Australasian ED, exploring potential factors correlated with variation.

**Methods:**

A retrospective multi-centre review of consecutive ED-ordered CTPA using standard radiology reports. ED CTPA report data were inputted onto preformatted data-sheets. The primary outcome was site level yield, analysed both intra-site and against a nominated 15.3% yield. Factors potentially associated with yield were assessed for correlation.

**Results:**

Fourteen radiology departments (15 ED) provided 7077 CTPA data (94% ≥64-slice CT); PE were reported in 1028 (yield 14.6% (95%CI 13.8–15.4%; range 9.3–25.3%; site variation p <0.0001) with four sites significantly below and one above the 15.3% target. Admissions, CTPA usage, PE diagnosis rates and size of PE were uncorrelated with yield. Large PE (≥lobar) were 55% (CI: 52.1–58.2%) and SSPE 8.8% (CI: 7.1–10.5%) of positive scans. CTPA usage (0.2–1.5% adult attendances) was correlated (p<0.006) with PE diagnosis but not SSPE: large PE proportions.

**Discussion/ Conclusions:**

We found significant intra-site CTPA yield variation within Australasia. Yield was not clearly correlated with CTPA usage or increased small PE rates. Both SSPE and large PE rates were similar to higher yield historical cohorts. CTPA use was considerably below USA 2.5–3% rates. Higher CTPA utilisation was positively correlated with PE diagnoses, but without evidence of increased proportions of small PE. This suggests that increased diagnoses seem to be of clinically relevant sized PE.

## Introduction

Much concern has been expressed about overuse of Computed Tomographic Pulmonary Angiography (CTPA) for pulmonary embolism (PE) diagnosis. [1–4] Emergency Department (ED) usage has been singled out both by other specialties and also Emergency Physicians.[1–6] CTPA has become the dominant modality for diagnosing PE, with imaging rates for PE increasing well ahead of population growth, whilst the reported rate of positive scans (yield) has decreased.[2,3]ED CTPA usage has increased markedly, with EDs now often being the largest single initiator of CTPA within their hospitals. [7–10]

The total numbers of diagnosed PE appear to have increased significantly with additional testing, but concerns have been raised that most of the additional diagnoses are small clinically unimportant PE, with significant numbers being false positives. [1–4] This concern is amplified by the use of newer generation, high resolution scanners where very small vessels and clots can be detected. [11] It has been argued that these scanners increase sub-segmental PE (SSPE) or isolated segmental PE diagnoses, where treatment benefits are controversial, and false positive rates may be high due to over-reporting. [1–6,8,10,11] Increased false positives are an expected outcome when an imperfect test is used more frequently in lower risk populations and false positive rates of up to 50% for SSPE have been reported. [1,5,6] Limited data suggests that newer CTs (16 slice or above) may be associated with higher proportions of SSPE(15–25% vs 5–8%) of all diagnosed PE.[6,11] The increased use of CTPA also subjects more patients to potential harms including radiation harms, contrast induced nephropathy (CIN) and allergies, as well as increasing costs and potentially delaying care. [1–4,5,7,11]

The concerns about CTPA overuse are largely driven by USA ED data, where studies over the last decade indicate PE yield may be routinely below 10% and CTPA is used in up to 3% of all adult attendances. [3,8,10,12–20] Data from other geographic regions is limited, but published yields, mainly from single centre studies, seem generally higher (12–20%) in Canada and Europe.[4,9,21–25] Data from Australasia is very limited in both quality and quantity, with yields reported from 6% to 14%. [26–28]

Although there is much commentary, few authors have attempted to define what a low or unacceptable yield is. Our group considered rates under 10% would clearly be too low. The British College of Radiologists have the only published target we could find (for use in audit), suggesting yield should be maintained over 15.3%.[29] Significant differences in practice and yield, particularly if also seen outside the USA, could indicate areas where future research to improve yield may be productive, clinically important and generalisable.[30]

Our aim therefore was to conduct a descriptive multicentre study of CTPA usage and yield (percentage of CTPA performed that are positive for PE) across Australasian EDs. We were especially interested in clinically significant variation in yield, what resolution scanners were being used, and to describe the proportions of PE of various sizes seen, particularly SSPE and larger PE (defined as lobar vessels or higher) rates.

The primary hypothesis was that there would be significant variability in CTPA yield across sites in Australasia. Differences of 6% or more were considered both clinically important and testable within available resources (see power calculation). Secondary aims included describing; the rates of SSPE (single or multiple PE at sub-segmental level); whether higher rates of SSPE are associated with the use of higher resolution CTPA; the distribution of PE at different levels of pulmonary vessels and whether lower yield and higher CTPA usage sites would be associated with increased proportions of small PE (particularly SSPE) and /or smaller proportions of large PE.

## Methods

This was a retrospective observational study conducted in 15 hospitals with accredited EDs across Australasia. Data were sourced from pre-existing radiology information systems, with additional demographic and radiological information fed back by staff from ED and radiology departments at each study site.

### Study site selection

Any site with an Australasian College for Emergency Medicine recognised ED was eligible to be involved and expressions of interest were via informal communication channels. Sites were only excluded if they could not provide consecutive data or could not identify ED patients for CTPA requests. We stopped additional sites joining when the minimal, central site personnel resources were becoming over-stretched.

### Case selection

Inclusions were consecutive CTPAs ordered from the ED for diagnosis of acute PE, with data including some part of the 2014 calendar year. However, sites could flexibly choose to run data collection forwards or backwards to recruit adequate numbers, depending on local access to data, and whether data had already been collected for other audits. Cases were excluded from further analysis if the study was not performed e.g. patient unco-operative, contrast extravasated or cancelled etc.; was not performed for detection of acute PE; not performed or ordered at the primary site, or not ordered in, or by, the ED; or where PE was found incidentally on another CT thorax protocol.

### Data collection

Clinical information was collected onto three preformatted Excelspreadsheets with a drop down menu using consistent nomenclature. Institutional data was collected for numbers of ED attendances and admissions; designated hospital /ED role; the generation/no. of slices of scanners used for CTPA; the preferred scanner and availability of scanning and whether VQ/ VQ SPECT was also available.

For each eligible CTPA, the formal radiology report was used as the definitive result. Data was collected on patient demographics (age/sex), whether a PE was definitively excluded or present, or if uncertainty was expressed in the report. If a PE was reported, but there was uncertainty expressed about scan quality, these were still included as a positive scan for this study. Reports with no documented caveats were assumed to be adequate studies. The highest level vessel with any clot seen in it as described in the formal radiology report was documented using the drop down menu. All sites collaborated with their radiology departments, and clarified issues with vessel nomenclature or numbering at the study site in discussion with the co-ordinating site. All data submitted centrally had to conform to the study nomenclature and be on standardised data collection sheets but with data quality control managed at the site level. Data on availability and use of VQ scanning at a site level was collected beforehand. A post-hoc survey was performed on whether sites had written formal diagnostic pathways available at the time of the study to examine if yield might have been effected by guideline availability.

### Definitions

Yield was defined as all CTPA reports with any acute PE stated in the radiology CTPA reports, as a proportion of all CTPA performed for acute PE from the ED. Sub-segmental PE was defined as any report where the highest level of PE reported was at the sub-segmental level, whether isolated or multiple. Small PE was defined as either SSPE (as above) or an isolated segmental clot. Large PE was defined as any PE described as being in a lobar or larger vessel. Adults were defined as any patient 18 years or older. Hospital admissions were defined as all admissions to another ward in the same hospital including observation ward admissions.

### Statistical analysis

#### Sample size calculation

Available literature reports CTPA yield ranging from 5 to 30%. [3,4,8–25] One small Australian ED study has a published yield of 6%. [6] Historical data from various regions report up to 25% yield in normal practice.[1–4] For a power of 80% and significance set at 5%, a sample size of 500 CTPA results per site was requested, allowing variations between sites of 5–6% to be detectable. This variation in yield was felt to be clinically important, well within reported variations in yield, and gave logistically feasible numbers of scans to review per site. Secondary outcomes were exploratory and separate power calculations were not performed for these.

#### Analysis of results

Summaries of continuous variables are reported as mean and standard deviation (SD). One sample binomial tests were used to compare the positive CTPA proportion at each site to a nominal threshold of 15.3%.[29] Univariate and multivariate logistic regression was used to compare CTPA yield, rates of SSPE and rates of large PE between sites. Multivariate analyses at site level were adjusted for both age and sex. Linear regression was used to explore the association between CTPA yield and admission rates, CTPA usage, PE diagnosed/1000 adult attendances rates of SSPE, small PE and large PE either as percentage of all CTPAs ordered, or percentage of only positive PE. Results were analysed using the R environment for statistical computing. ([31]

### Ethics

The co-ordinating site, Sir Charles Gairdner Hospital (SCGH) gained initial ethics approval for the study as low risk quality improvement research, with all other sites gaining either state based or local ethics approval based on the index ethics approval. Consent was waived due to the low risk nature of the study and use of de-identified data.

## Results

### Site demographics /characteristics (Table 1)

**Table 1:** Site ED characteristics, collection dates and CT types used.

Site	ED type	Region	ED Adult attendances during study (per. year)	CT(s) used- slice	Dates data collected
**A**	Mixed- outer metro	WA	67600 (44700)	64*/128	1/13-6/14
**B**	Major tertiary–metro mixed	Victoria	50100 (42100)	128/320*	4/13-6/14
**C**	Major tertiary -regional	Qld	110000 (57900)	256/320*x2	8/12-6/14
**D**	Outer metro mixed	Qld	84800 (42400)	128x2	7/12-6/14
**E**	Tertiary/outer metro mixed	Victoria	83300 (100000)	64*/256*/ 320	2/14-11/14
**F**	Regional- mixed secondarysecondary	NZ	231900 (14500–20500)	16	10/01-6/14
**G**	Major tertiary adult	Qld	44800 (59700)	64/128x2 /256*x2	10/13-6/14
**H**	Major tertiary adult	Qld	70200(70200)	64/128*/256	7/13-6/14
**I**	Mixed major—tertiary	NSW	37600 (56400)	64/256x2*	7/14-2/15
**J**	Major tertiary—adult	WA	80300 (80300)	64*/128x2	7/13-6/14
**K**	Major tertiary adult	WA	128000 (64000)	64/320*	2/12-1/14
**L**	Major tertiary mixed	Qld	33900 (50800)	64/256*x2 /320	11/13-6/14
**M**	Outer metro- mixed	NSW	38700 (38700)	80	7/13-6/14
**N**	Major referral -regional mixed	Qld	60800 (60800)	64/256*	1/14-12/14

*Main scanner used for CTPA. WA = Western Australia, Qld- = Queensland, NZ- New Zealand, NSW–New South Wales

Fourteen reporting sites (but 15 EDs, as two ED had centralised reporting as a single unit) provided consecutive data for 7077 ED ordered CTPAs. Numbers ranged from 324 to 1057 CTPA per site, over 8 months to 2 year periods(from 01/2012 to 02/2015), with the exception of site F which provided consecutive data from twelve years of CTPA use (from 2002), due to lower usage rates in a smaller adult ED population. All sites used 64 slice or higher CT scanners, except F (16 slice at all times). Sites were varied in their characteristics including: multiple states and countries; tertiary vs non-tertiary hospitals; central, outer metropolitan and regional sites; adult only vs. general populations; and considerable variation in admission rates (Table 2) from the ED.

**Table 2:** ED attendances, admits, yield; CTPA usage and PE diagnosis per 1000 ED adults.

Site	Adult ED patients during study	ED Admits during study (%)*	CTPA (n) per site	YIELD—% +ve PE	CTPA/ 1000 ED adults	CTPA/ 1000 ED admits	CTPA +ve for PE /1000 ED adults^$^
A	67601	9379 (13.9%)	520	15.8	7.7	55.4	1.2
B	50120	13547 (56.4%)	499	13.4	10.0	36.8	1.3
C	109942	54963 (42.3%)	501	16.0	4.6	9.1	0.7
D	84800	44000 (33%)	515	9.3	6.1	11.7	0.6
E	83300	39600 (54.5%)	507	16. 6	6.1	12.8	1.0
F*	232000	71100 (34%)	443	25.3	1.9	6.2	0.5
G	44795	20830 (46.7%)	499	17.0	11.1	24.0	1.9
H	70209	23450 (33.2%)	359	10.0	5.1	15.3	0.5
I	37643	20686 (40.9%)	324	16.0	8.6	15.7	1.4
J	80326	37392 (46.5%)	491	12.4	6.1	13.1	0.8
K	129000	74300 (58%)	1053	16.3	8.2	14.2	1.3
M	38656	13575 (26.1%)	420	12.6	10.9	30.9	1.4
L	33897	25179 (38.1%)	498	9.8	14.7	19.8	1.0
N	60793	21018 (29.3%)	435	11.7	7.2	20.7	0.8
Totals OR Means* **(CI)**	1140030	459080 (40.3%)	7064	14.3*(13.8–15.4%)	6.2*	15.4*	0.9*^$^

$ NB that some sites (12/14) also use VQ for a small proportion of their patients in the assessment for possible PE so that the rate of PE/1000 will be an under-estimation of total population diagnosis

Of the 7064 CTPA with complete data, 3871 were performed in females (54.8% vs 45.2% males), p<0.0001 for difference of 9.6% (CI 7.95–11.25%)) and the mean age was 60.0 years (CI 59.6–60.4, SD 16.65). Yield was significantly lower amongst females (12.1%; CI 11.3–12.9%) than males (17.6%; CI 16.7–18.5%); p < 0.0001 for difference in proportions. The mean age of those with a positive scan was 61.5 (SD = 15.6) vs. 59.7 (SD = 16.7) years for negative scans (p = 0.0009 for difference).

### Outcomes

The overall yield for all CTPA performed at the 14 reporting sites was 14.6% (95% CI: 13.8–15.4%), ranging from 9.3 to 25.3% (p<0.0001 for overall differences across all sites). (Table 2) The lowest three sites by yield were significantly lower in pairwise comparisons than the seven sites with the highest yield. In addition, site F had significantly higher yield than all other sites with their yield remaining within a 3% range of 25% throughout the 12 years. Against the suggested hypothesised yield of 15.3%, four sites had significantly lower rates of yield (D, H, L, N) and one site was significantly higher (F).[29] A sensitivity analysis removing site F results found no difference to any of the outcomes reported. Only four sites (C,D,F and M) did not have formal diagnostic pathways during the study period (including risk stratification tools and direction on use of testing), with yield for these sites spread across the range of results.

#### SSPE/ small PE

**Fig 1:**
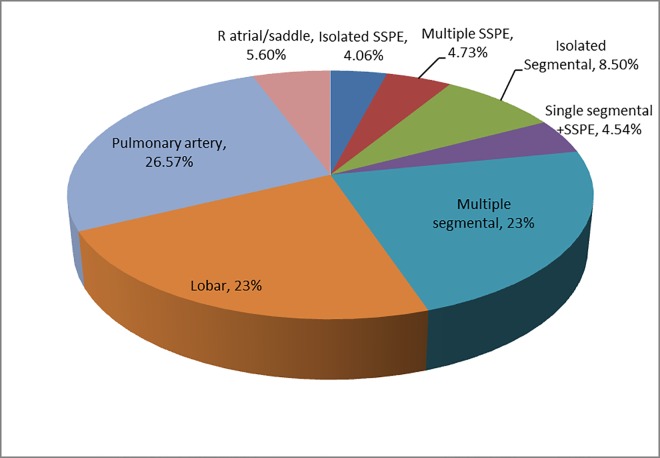
Rates of PE at different levels in total population of positive PE on CTPA (%).

Table 3 and Fig 1. SSPE (isolated or multiple) were 8.8% (CI 7.1–10.5%) of all diagnosed PE with prevalence ranging from 2.0 to 15.8% of diagnosed PE, with only two marginally significant differences on pairwise comparisons. Variation in small PE prevalence ranged from 10.8 vs 21.1% and no comparisons were significantly different. Variation in the rates of diagnosed SSPE as a proportion of all CTPAs performed ranged from 0.2–2.5% between sites, and small PE from 0.8% to 3.2%. Some differences were marginally significant but consistent with expected statistical variation when performing multiple comparisons.

**Table 3:** SSPE/ Large PE rates as % of positive AND total CTPA (small PE only as % of positive CTPA).

SITE	All +ve PE on CTPA n	YIELD% +ve PE	SSPE n as (%)of +ve CTPA	Small PE n as (%) of +ve CTPA	Large PE n as (%) +ve CTPA	SSPE as %of all CTPA	Large PE as % of all CTPA
A	82	15.8	13 (15.8)	16 (19.5)	45 (54.8)	2.5	8.6
B	67	13.4	5 (7.5)	14 (20.9)	26 (38.8)	1.0	5.2
C	80	16.0	6 (7.5)	14 (17.5)	50 (62.5)	1.2	10.0
D	48	9.3	6 (12.5)	25 (52.1)	25 (52.1)	1.1	4.8
E	84	16. 6	8 (9.4)	56 (65.9)	56 (65.9)	1.6	11.0
F	112	25.3	6 (5.4)	68 (60.7)	68 (60.7)	1.3	15.3
G	85	17.0	6 (7.1)	35 (41.2)	35 (41.2)	1.2	7.0
H	36	10.0	1 (2.7)	19(51.3)	19 (51.3)	0.3	5.2
I	52	16.0	2 (3.8)	11 (21.1)	22 (42.3)	0.6	6.75
J	61	12.4	8 (13.1)	11 (18)	36 (59.0)	1.6	7.3
K	172	16.3	22 (12.8)	33 (19.2)	103 (59.9)	2.1	9.7
L	49	12.6	1 (2.0)	5 (10.2)	26 (53.1)	0.2	5.2
M	53	9.8	2 (3.7)	11 (20.4)	31 (57.4)	0.5	7.3
N	51	11.7	7 (13.7)	29 (56.9)	29 (56.9)	1.1	6.6
**Totals(95% CI)**	**1028**	**14.3%(13.8–15.4)**	**179(8.8% CI:7.1–10.5)**	**179(17.3% CI:15.0–19.6)**	**571(55.2% CI:52.1–58.2)**	**1.3%(CI 1.0–1.5)**	**8.1%(CI 7.4–8.7)**

NB totals do not include all PE as intermediate (non-small-non large) PEs not included.

#### Lobar or higher level PE (large PE)

**Fig 2:**
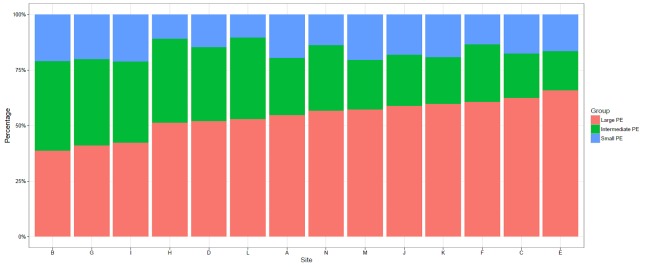
Proportion of small, intermediate and large PE as % of all PE at each site.

**Fig 3:**
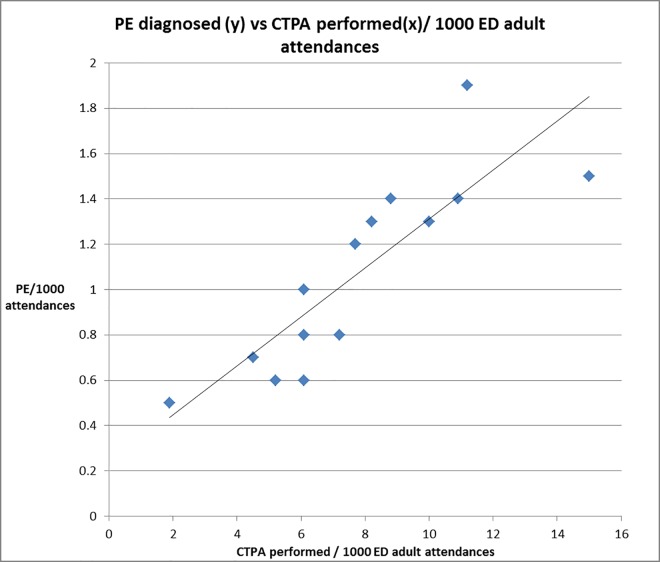
CTPA utilisation vs no. of PE diagnoses per 1000/ ED adult attendances.

**(Figs 2 and 3, Table 3)** Large PE comprised 55.2% (CI 52.1–58.2%) of all PE reported on CTPA. At a site level, the rates of large PE as a percentage of all positive CTPA ranged from 38.8 to 66.0% and as a percentage of all CTPA performed ranged from 4.8 to 15.3% (with 13 sites from 4.8–11%, site F, 15.3%). Site F was significantly different from all other sites in all pairwise comparisons either as a proportion of positive PE or all CTPA performed. A third of all pairwise comparisons were significant for large PE as a proportion of all CTPA performed.

### Other potentially important correlations/ associations

We assessed for correlations between yield per site and rates of SSPE, small PE and large PE either as percentage of all CTPAs ordered, or percentage of only positive PE when correlated against yield. The only significant association was that with increasing CTPA yield, increasing numbers of both small and large PE were seen (but not SSPE alone) as a percentage of all CTPA performed. This correlation was not seen when the small or large PE were considered as a proportion of positive CTPA against yield. There was also no significant correlation between CTPA yield and rates of CTPA usage, defined as number of CTPA performed per 1000 adult attendances to ED (data Table 1). There was however a positive linear correlation (r = 0.686, p = 0.006) between rates of CTPA usage and rates of PE diagnosed per 1000 adult attendances. (Fig 2)

## Discussion

### Key findings

This is the largest published study we are aware of specifically looking at CTPA yield and site variation, particularly for newer high resolution CT (94%, 64 slice or greater) We found significant variation in yield, from 9.3% to 25.3%, with four sites significantly below a hypothesised acceptable rate of 15.3%.[29] Sites were very different (as designed) for many demographic and role delineation issues. Age and male sex were significantly associated with positive CTPA. Other factors previously suspected of affecting yield such as CTPA usage rates (range 2–15 CTPA per 1000 adult ED attendances) or admission rates (range 13–58%, used as a marker of complexity) were not significantly correlated with site yield in this region.

The overall proportion of SSPE was 8.8%, ranging from 2.0–15.9% of all PE diagnosed, but the more inclusive small PE grouping found no significant variation (10.2–21.2%). The statistically different SSPE rates between sites were within expected variation for multiple pairwise comparisons. As a proportion of all CTPA, small PE rates increased with yield, as did large and intermediate (non-large, non- small) PE e.g. as more PE were diagnosed, all sizes of PE seemed to be diagnosed more frequently (Fig 3). Large PE (lobar vessels or larger), were 55% of all PE, with significant inter-site differences, but without significant correlation to either site yield or PE /1000 adult ED attenders. Finally a significant linear correlation was seen between CTPA use per 1000 adult ED attendances and PE diagnosis rates. (Fig 2)

### Comparison of key findings with previous literature

Our primary outcome was to observe for variation in site yield, particularly when using newer high resolution CTPA, and to examine factors that might potentially explain some of those differences. Practice variation is associated with poor patient outcome and excessive resource use, and is suggested as an important area for targeted audit, interventions and research. [30] Concern about variable practice and excessive CTPA use has been raised repeatedly in the literature, although very low rates of CTPA yield (<10%) are rarely described outside of the USA.[1–6,8–25] Our study found yields occasionally dropped just below 10% (2/14 sites), but 50% of sites had yields below the suggested UK target of 15.3%, with an overall population yield of 14.3% (upper CI:15.4%).[29] A recent systematic review of strategies to improve CTPA yield and reduce radiological testing suggested improvements of 3–5% in yield should be readily achievable. [32] Our data suggests that our region may represent an area where improvement in CTPA yield and reduced CTPA usage may be achievable.

As described in other populations, CTPA was ordered more frequently in females (an almost universal finding), but more unusually males had significantly higher rates of PE diagnosed. [3,33–36] Age and sex were included in the analysis of yield variation at site level. We found no correlation between lower site yield with increasing CTPA usage although it has been strongly suggested as a driver of low yield, mainly based on USA data.[1–4]However it is important to note that USA sites often report higher CTPA utilisation rates (20–35 per thousand adult ED attendances) than we describe here (2-15/1000). [8,23,35] It is possible there is a threshold utilisation rate beyond which yield drops dramatically without additional significant diagnoses, therefore providing minimal patient benefit, but significant increased harms.

One of the key concerns about excessive CTPA use and low yields are that small PE, particularly SSPE, may be diagnosed more often when using newer high resolution CT scanners, with many of these potentially being false positives.[1–4] These concerns were not confirmed in this study with an overall SSPE rate of 8.8%. Our rate is similar to Carrier et als’ recent meta-analysis findings for previous generations of lower resolution multi-slice scanners (2–16 slice, approximately 7%), including the seminal PIOPED2 study.[11,33] Indeed our SSPE rate overlaps with Carrier’s reported rate for prospective single slice CTPA studies, and SSPE rates seen on pulmonary angiography in the PIOPED 1 study. [11,33,37] However our findings cannot preclude increased rates of SSPE being an issue in other reporting environments or if CTPA usage increased to USA levels. Two recent studies specifically reviewed positive scans using specialist cardiothoracic radiologist reviews found rates of SSPE of 18% (yield 19%- Hutchinson, Eire) and 26% (yield 9%, Miller, USA).[6,38] Both studies found very high rates of probable or definitive false positive diagnoses (56% and 42% respectively) amongst CTPA reported as isolated SSPE or isolated segmental PE. It seems likely that there are differences between sites and regions in radiology reporting practice, with institutional or regional willingness to “miss” PE potentially explaining some of this variation.

It has also been suggested that with excessive CTPA use, larger, and more clinically important PE, would become a significantly smaller proportion of all PE i.e. most of the additional diagnoses would be smaller clots.[1–4,6,31,39] However, our study found an overall rate of large PE of 55%, similar to rates reported from the two seminal PIOPED studies (PIOPED1-56% and PIOPED 2–62%) even though their yields were significantly higher than ours e.g. 27% in PIOPED1, 23% in PIOPED2.[33,37] Morley et al recently published a decade of single institution data finding that imaging-for PE increased 250% (>90% CTPA), PE diagnoses increased 160% and yield reduced from 24 to 20%, but the proportion of large PE was maintained.[40] They suggested that increased imaging in their institution was finding increased rates of previously undiagnosed but clinically significant PE.[40] Our study found no significant correlation for site yield or CTPA utilisation vs. the proportion of large PE. Our data suggests that, within this regions current rates of CTPA utilisation (up to 1.5% of adult ED attenders getting CTPA) and yield, proportions of large PE seem to be maintained at historical levels. It is possible that if utilisation rates doubled and yield routinely dropped to well below 10%, as seems to be the case in significant parts of the USA, that small: large PE ratios may change.[3,38,39]

Finally as noted above CTPA use in our cohort of EDs (2-15/1000) seems to be considerably less than reported USA rates of 20-40/1000 ED attendances. [8,23,35] Variations in CTPA utilisation showed no association with yield, or with size of PE, but were strongly positively correlated with increased rates of PE per 1000 adult attendances (Fig 2). This, alongside the stable small and large PE rates (compared with historical data), suggests that additional PE’s diagnosed with increased CTPA use in our region would seem to be clinically important.

### Study weaknesses

The study relied on retrospective data collected from routine clinical practice. Interpretation of the radiology reports although standardised against clear criteria could still have interpretative errors which would need to be assessed by testing inter-rater reliability. However interpretative errors would not be expected to change outcomes with any particular bias. Additionally clinicians rely on standard radiology reporting to manage their patients in routine clinical practice. Resources did not allow detailed on site reviews of data collection quality, although any significant issues, nomenclature problems or evidence of non-consecutive data at any site were discussed, reviewed and resolved between site principal investigators and the co-ordinating investigator(DM). It would have been preferable to have similar timing for all sites recruitment, the exact same numbers of CTPA per site, and slice used for each patient but this was not logistically possible. However, no site changed scanner type during their collection periods and all sites (except F) collected data on 64 slice or higher CTPA within a 3 year period from 2012–2015. Detailed data about patient selection, screening, risk stratification and D-Dimer testing would also have been useful in looking at issues around yield, but this was logistically implausible with limited resources. However all but four sites had access formal diagnostic pathways available to clinicians, and those four sites had yields across the reported range. Although our data is limited to ED ordered CTPA, this area has had the most concerns expressed re excessive use, poor yield and patient harms, both by ED physicians and other specialists. [1–6]Finally detailed data on how many patients had VQ scans performed (where available) would have been preferable. However although VQ scanning was available at all but two sites, no site used it as their primary investigation, and a large effect on overall CTPA yield or PE diagnosis rates would be unexpected. Finally, we have no specific information on variability in radiology reporting practices or accuracy at different sites.

### Study strengths

This is a large study of CTPA yield and utilisation from diverse sites giving significant new data about real world reporting and utilisation practices when using high resolution CT scanners. The use of multiple sites with varied outcomes and yield has allowed important correlations and concerns about CTPA use to be explored. The relative ease of collecting such large amounts of readily available data suggests the possibility of regular audit, registry data and prospective research for these outcomes in everyday practice. The large number of centres with varied practice settings suggest these findings have external generalisability for other regions.

### Future research

More detailed research should examine factors driving yield and usage at a local level; whether current guidelines to avoid CTPA are being implemented; and if so, are they working. It is possible to envisage either randomised trials at multiple sites or with cluster design, implementing strategies such as enforced risk stratification, routine D-Dimer use and senior clinician review to improve yield and/ or reduce CTPA usage rates.[32] Additionally, given the variation in the sizes of PE reported, it would be useful to compare different yielding sites images against gold standard thoracic radiologist reviews to assess for systematic reporting biases. Longitudinal studies at multiple sites could examine changes in yield, CTPA usage and PE diagnosis rates, or other important outcomes, over time.

## Conclusions

This large multiple site study of yield from modern high resolution CTPA, found significant variation between sites, with half the sites having yields below a hypothesised “acceptable” rate of 15.3% yield. Lower yields with high resolution CTPA were not significantly associated with increased ratios of SSPE/small PE vs. large PE with similar proportions of both SSPE and large PE to historical cohorts. Increasing use of CTPA was associated with increased rates of PE diagnosis, with the additional PE not being obviously smaller or clinically inconsequential in this cohort.
