# Correction: RESPECT-ED: **R**ates of Pulmonary **E**mboli (PE) and **S**ub-Segmental **PE** with Modern **C**omputed **T**omographic Pulmonary Angiograms in **E**mergency **D**epartments: A Multi-Center Observational Study Finds Significant Yield Variation, Uncorrelated with Use or Small PE Rates

**DOI:** 10.1371/journal.pone.0184219

**Published:** 2017-08-29

**Authors:** David Mountain, Gerben Keijzers, Kevin Chu, Anthony Joseph, Catherine Read, Gabriel Blecher, Jeremy Furyk, Chrianna Bharat, Karthik Velusamy, Andrew Munro, Kylie Baker, Frances Kinnear, Ahses Mukherjee, Gina Watkins, Paul Buntine, Georgia Livesay, Daniel Fatovich

Although there are multiple corrections to both formatting and some content in Tables and Figures, and some minor changes to reported results and analysis, none of these corrections alter any major findings, discussions or conclusions from the initial study.

There are a number of errors in Table 2. Row M should appear after Row L. The values in the column “Adult ED patients during study” are incorrect. “ED Admits during study (%)*” values are incorrect in rows B, C, E, F, and L. “CTPA (n) per site” values are incorrect in rows C, E, H, K, L, M, and N. The “CTPA/ 1000 ED admits” and “CTPA +ve for PA /1000 adults^$^” values in row L are incorrect. Please see the corrected Table 2 below. Totals and means have been recalculated, as necessary.

**Table 2:** ED attendances, admits, yield; CTPA usage and PE diagnosis per 1000 ED adults.

Site	Adult ED patients during study	ED Admits study (%)*	CTPA (n) per site	YIELD—% +ve for PE	CTPA/1000 ED adults	CTPA/ 1000 ED admits	CTPA +ve for PE /1000 ED adults^$^
A	67601	9379 (13.9%)	520	15.8	7.7	55.4	1.2
B	50120	28100 (36.4%)	499	13.4	10.0	17.8	1.3
C	109942	54963 (35.2%)	500	15.8	4.6	9.1	0.7
D	84800	44000 (33%)	515	9.3	6.1	11.7	0.6
E	83300	47800 (54.5%)	509	16.7	6.1	10.6	1.0
F*	232000	71100 (22.8%)	443	25.3	1.9	6.2	0.5
G	44795	20830 (46.7%)	499	17.0	11.1	24.0	1.9
H	70209	23450 (33.2%)	362	10.2	5.2	15.4	0.5
I	37643	20686 (40.9%)	324	16.0	8.6	15.7	1.4
J	80326	37392 (46.5%)	491	12.4	6.1	13.1	0.8
K	129000	74300 (58%)	1056	16.2	8.2	14.2	1.3
L	33897	17200(38.1%)	500	9.8	14.7	29.1	1.4
M	38656	13575 (26.1%)	421	12.8	10.9	31.0	1.4
N	60793	21018 (29.3%)	436	11.7	7.2	20.7	0.8
Totals OR Means* **(CI)**	1123082	483793	7075	14.6* (13.8–15.4%)	6.3*	14.6*	0.9*^$^

^$^ NB that some sites (12/14) also use VQ for a small proportion of their patients in the assessment for possible PE so that the rate of PE/1000 will be an under-estimation of total population diagnoses

There are multiple errors in Table 3. The “Large PE n as (%) +ve CTPA” values are incorrectly duplicated for the “Small PE n as (%) of +ve CTPA” values in rows D, E, F, G, H, and N. The “Totals (95%CI)” value in the column “SSPE n as (%) of +ve CTPA” is incorrectly duplicated in the “Small PE n as (%) of +ve CTPA” column. “Totals (95%CI)” in column “Yield % +ve PE” is incorrect. There are minor discrepancies throughout the table in comparison to the source data. Please see the corrected Table 3 below. Totals and means have been recalculated, as necessary.

**Table 3:** SSPE/ Large PE rates as % of positive AND total CTPA (small PE only as % of positive CTPA).

SITE	All +ve PE on CTPA n	Yield % +ve PE	SSPE n as (%) of +ve CTPA	Small PE n as (%) of +ve CTPA	Large PE n as (%) +ve CTPA	SSPE as % of all CTPA	Large PE as % of all CTPA
**A**	**82**	15.8	13 (15.9)	16 (19.5)	45 (54.9)	2.5	8.7
**B**	**67**	13.4	5(7.5)	14(20.9)	26(38.8)	1.0	5.2
**C**	**79**	15.8	6 (7.6)	14 (17.7)	50 (63.3)	1.2	10.0
**D**	**48**	9.3	6(12.5)	7(14.6)	25(52.1)	1.2	4.9
**E**	**85**	16.7	8(9.4)	14(16.5)	56(65.9)	1.6	11.0
**F**	**112**	25.3	6(5.4)	15(13.4)	68 (60.7)	1.3	15.3
**G**	**85**	17.0	6(7.1)	17 (20.0)	35(41.2)	1.2	7.0
**H**	**37**	10.2	1(2.70)	4(10.8)	19 (51.4)	0.3	5.2
**I**	**52**	16.0	2 (3.8)	11(21.1)	22(42.3)	0.6	6.8
**J**	**61**	12.4	8 (13.1)	11(18.0)	36(59.0)	1.6	7.3
**K**	**171**	16.2	22(12.9)	33(19.3)	102(59.6)	2.1	9.7
**L**	**49**	9.8	1(2.0)	5(10.2)	26 (53.1)	0.2	5.2
**M**	**54**	12.8	2(3.7)	11(20.4)	31 (57.4)	0.5	7.4
**N**	**51**	11.7	5(9.8)	7(13.7)	29(56.9)	1.1	6.6
**Totals** **(95%CI)**	**1033**	14.6*	**91 (8.8%)** **(CI:7.1–10.5%)**	**179 (17.3%)** **(CI: 15.0–19.6%)**	**570 (55.2%)** **(CI 52.1–58.2%)**	**1.3%** **(CI1.0–1.5%)**	**8.1%** **(CI: 7.4–8.7%)**

NB totals do not include all PE as intermediate (non-small-non large) PEs not included.

Fig 3 contains two data point errors. Please see the corrected Fig 3 here.

**Fig 3:**
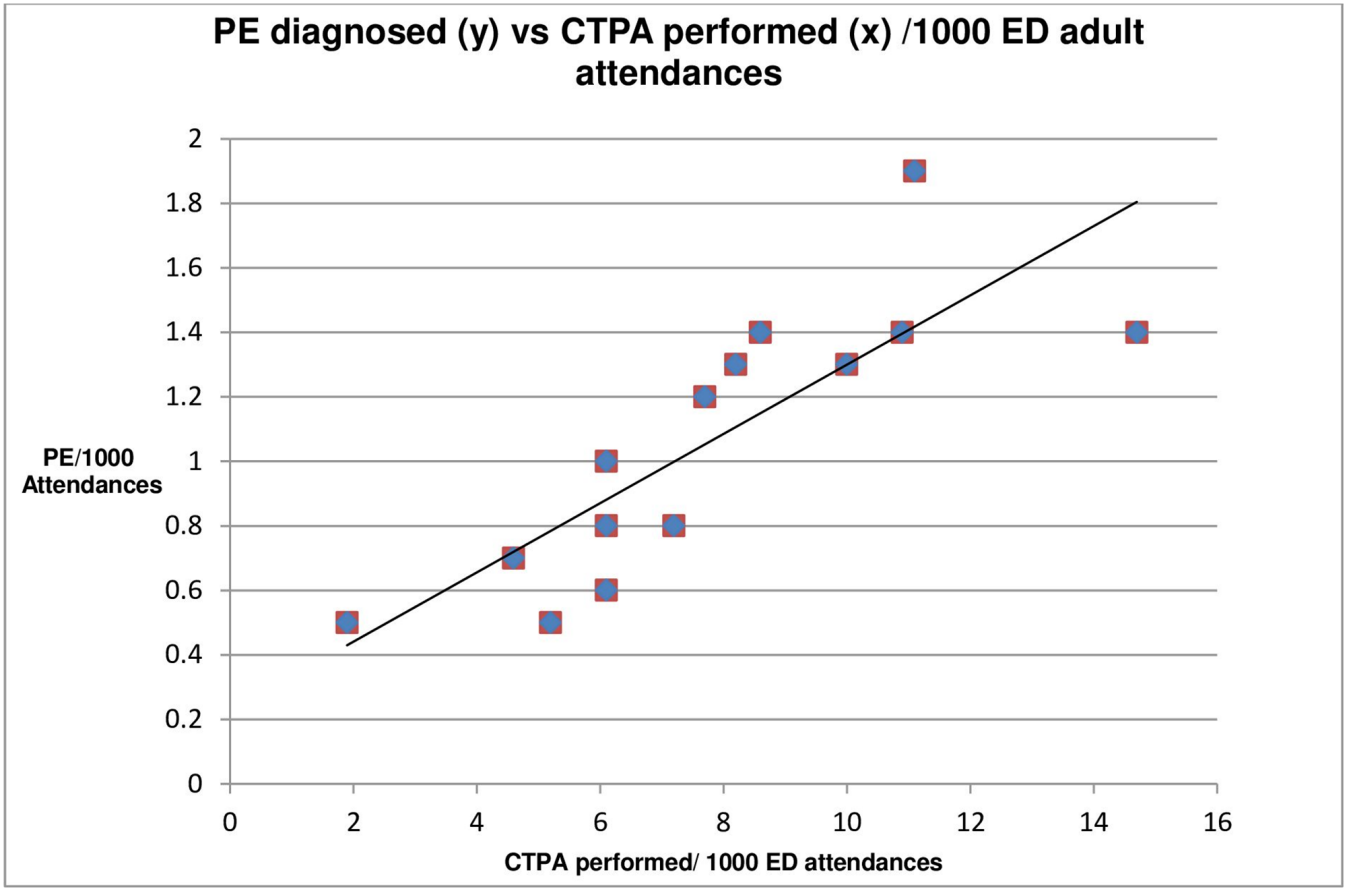
CTPA utilisation vs no. of PE diagnoses per 1000/ ED adult attendances.

There are a number of errors throughout the article. In the Abstract, the first sentence of the Results section should read: Fourteen radiology departments (15 ED) provided 7075 CTPA data (94% ≥64-slice CT); PE were reported in 1033 (yield 14.6% (95%CI 13.8–15.4%; range 9.3–25.3%; site variation p <0.0001) with four sites significantly below and one above the 15.3% target.

The first two sentences of the “Site demographics /characteristics” section of the Results should read: Fourteen reporting sites (but 15 EDs, as two ED had centralised reporting as a single unit) provided consecutive data for 7075 ED ordered CTPAs. Numbers ranged from 324 to 1056 CTPA per site, over 8 months to 2 year periods (from 01/2012 to 02/2015), with the exception of site F which provided consecutive data from twelve years of CTPA use (from 2002), due to lower usage rates in a smaller adult ED population.

The second paragraph of the same section should read: Of the 7062 CTPA with complete demographic data, 3870 were performed in females (54.8% vs 45.2% males), p<0.001 for difference of 9.6% (CI 7.95–11.25%)) and the mean age was 60.0 years (CI 59.6–60.4, SD 16.6). Yield was significantly lower amongst females (12.1%; CI 11.3–12.9%) than males (17.6%; CI 16.7–18.5%); p < 0.0001 for difference in proportions. The mean age of those with a positive scan was 61.5 (SD = 15.6) vs. 59.7 (SD = 16.7) years for negative scans (p <0.001 for difference).

The paragraph of the “SSPE/ small PE.” section of the Results should read: Table 3 and Fig 1. SSPE (isolated or multiple) were 8.8% (CI 7.1–10.5%) of all diagnosed PE with prevalence ranging from 2.0 to 15.9% of diagnosed PE, with only three marginally significant differences on pairwise comparisons. Variation in small PE prevalence ranged from 10.8 vs 21.1% and no comparisons were significantly different. Variation in the rates of diagnosed SSPE as a proportion of all CTPA’s performed ranged from 0.2–2.5% between sites, and small PE from 1.0% to 3.4%. Some differences were marginally significant but consistent with expected statistical variation when performing multiple comparisons.

The last sentence in the “Other potentially important correlations/ associations” section of the Results should read: There was however a positive linear correlation (r = 0.822, p = <0.001) between rates of CTPA usage and rates of PE diagnosed per 1000 adult attendances.

The first sentence of the second paragraph in the “Key findings” section of the Discussion should read: The overall proportion of SSPE was 8.8%, ranging from 2.0–15.9% of all PE diagnosed, but the more inclusive small PE grouping found no significant variation (10.2–21.1%).

The fourth sentence of the “Comparison of key findings with previous literature” section of the Discussion should read: Our study found yields occasionally dropped just below 10% (2/14 sites), but 50% of sites had yields below the suggested UK target of 15.3%, with an overall population yield of 14.6% (upper CI:15.4%).
